# Electrospun Superhydrophobic Silica Nanofiber Coatings for Enhanced Pool Boiling on Copper Foam

**DOI:** 10.3390/nano16171048

**Published:** 2026-08-22

**Authors:** Sun Liya, Lang Zhongmin, Yu Ying

**Affiliations:** College of Chemical Engineering, Ordos Institute of Technology, Ordos 017000, China

**Keywords:** pool boiling, micro/nanocomposite structures, HCFs, electrostatic spinning method

## Abstract

Superhydrophobic SiO_2_ nanofibers were deposited on copper foam substrates via micro/nano surface modification to improve the pool boiling heat transfer performance of porous copper media. By adopting an electrospinning technique, uniform and robust superhydrophobic SiO_2_ nanofibers were firmly deposited on copper foam skeletons, forming interconnected porous structures with intrinsic superhydrophobic characteristics. The fabricated superhydrophobic nanofiber structures greatly reduce bubble nucleation resistance and provide sufficient stable vaporization sites, effectively promoting boiling heat transfer enhancement. Experimental results verify that surface modification with superhydrophobic SiO_2_ nanofibers significantly improves the overall boiling performance of copper foam. The sample with a nanofiber loading of 1.8 mg achieves the optimal thermal performance, presenting lower wall superheat, higher critical heat flux, and an improved heat transfer coefficient. CFD simulations were conducted, and the numerical results exhibit good consistency with experimental measurements.

## 1. Introduction

With the rapid upgrading of new energy storage systems and industrial heat exchange equipment, the system heat flux density has been continuously improved, leading to a growing heat dissipation load on core components. Accordingly, high-efficiency and low-energy-consumption heat transfer technologies have become the core technical support for the long-term and stable operation of thermal equipment [1,2,3,4]. Pool boiling heat transfer has been widely applied in electronic component thermal management and industrial waste heat recovery by virtue of its excellent comprehensive performance, including high heat transfer capacity, no need for complex structural reconstruction, passive operation independent of external driving energy, and superior long-term operational stability [5,6,7,8]. As one of the key characteristic parameters of pool boiling, the onset of nucleate boiling (ONB) plays a decisive role in the start-up characteristics and low-temperature heat transfer performance of thermal systems [9]. A lower wall superheat can induce rapid boiling phase transition and significantly enhance the heat dissipation capacity under small temperature differences, which has important engineering significance for reducing the energy consumption of boiling heat transfer equipment [10].

Traditional smooth metal heat transfer surfaces have inherent defects in thermal performance. Smooth substrates such as pure copper and pure aluminum present a single surface wettability state with sparse active nucleation sites, which easily causes excessive ONB superheat and delayed thermal response during boiling processes [11]. Moreover, smooth surfaces are susceptible to sharp wall temperature rises and premature boiling crises under high heat flux conditions, which fail to meet the low-temperature and high-efficiency heat dissipation requirements of modern high-heat-flux thermal equipment [12,13,14,15].

Sitar [16] investigated the pool boiling heat transfer performance of etched textured silicon surfaces. Experimental results showed that the etched silicon sample with a pore diameter of 30 μm and a pore spacing of 0.125 mm achieved a maximum heat transfer coefficient (HTC) enhancement of 244%. Shree [17] analyzed the boiling characteristics of copper substrates modified by TiO_2_ spray coating. Compared with pristine smooth copper surfaces, the TiO_2_ plasma-sprayed coating increased the critical heat flux (CHF) by 47–58%. In addition, Salar [18] prepared graphene oxide coatings on copper substrates via the spin-coating method and systematically evaluated their pool boiling performance. The test results indicated that the coating prepared with a concentration of 4 mg·mL^−1^ exhibited the optimal heat transfer performance, with maximum CHF and HTC improvements of 59% and 84.7%, respectively.

Nevertheless, existing surface modification methods including etching, spraying, and thin-film deposition still have unavoidable technical limitations. Etching treatment is prone to damaging the metal substrate structure, with poor processing stability and controllability [19,20,21]. Spray coatings suffer from low interfacial bonding strength and are prone to peeling failure under high-temperature boiling environments [22,23,24]. Furthermore, conventional deposition technologies can only form single and homogeneous interfacial structures, which cannot realize the structural matching and adaptive design between microscale pore architectures and macroscopic substrates [25,26,27]. Therefore, the development of innovative surface modification technologies with simple preparation processes, stable structural properties, and excellent thermal performance has become a research hotspot in the field of pool boiling heat transfer enhancement.

As a typical three-dimensional (3D) porous metal functional material, copper foam possesses interconnected pore channels, a large specific surface area, excellent thermal conductivity, and outstanding mechanical stability. Compared with compact solid metal surfaces, its unique porous structure can greatly increase the density of bubble nucleation sites, making it an ideal substrate for high-performance pool boiling heat transfer [28,29]. However, pristine copper foam presents intrinsic superhydrophilicity, which enables the cooling liquid to spread evenly on the surface. This inherent property leads to high ONB superheat and unsatisfactory low-temperature heat transfer efficiency, severely restricting its engineering application in precise low-temperature heat dissipation scenarios. Despite the progress achieved in previous copper foam modification for pool boiling enhancement, several notable research gaps remain to be addressed. First, conventional surface modification approaches tend to form dense coatings that block the interconnected porous architecture of copper foam, restricting bubble escape and liquid rewetting and capping further improvements in boiling heat transfer performance. Second, conventional silica coatings are formed by simple particle stacking, which yields weak interfacial bonding and inferior thermal stability; such coatings are susceptible to peeling and failure under long-term boiling thermal cycles. Third, most existing investigations place emphasis primarily on macroscopic boiling performance promotion, yet lack systematic multi-scale mechanistic analysis linking microstructural features, interfacial wettability, and critical boiling heat transfer parameters.

To solve the above technical bottlenecks, this work adopts electrospinning technology to fabricate a superhydrophobic silica nanofiber composite coating on 3D porous copper foam substrates, realizing the efficient and controllable construction of hierarchical micro/nanocomposite heat transfer interfaces. Different from conventional particle-deposited and hydrothermal-modified copper foam surfaces that suffer from severe pore blockage, uneven deposition, and poor interfacial stability, the continuous electrospun nanofiber network enables conformal wrapping on complex 3D pore surfaces while preserving the intrinsic open pore channels of the substrate. The proposed strategy exhibits distinct superiorities in preparation technology, microstructure design, and thermal performance optimization. In terms of the manufacturing process, electrospinning involves simple operation and tunable structural parameters [30,31]. Benefiting from conformal nanofiber wrapping characteristics, this technique effectively avoids common defects of traditional modification methods, such as uneven microstructure distribution and easy coating peeling under thermal cycling, thus improving the process repeatability and engineering practicability of surface modification.

In terms of structural design, the integration of porous microscale copper foam skeletons and interwoven silica nanofiber networks constructs a unique micro/nano hierarchical pore structure. This structure effectively improves the surface roughness of heat transfer interfaces and creates abundant, uniformly distributed, high-efficiency bubble nucleation sites. The rationally designed superhydrophobic micro/nanocomposite structure optimizes solid–liquid interfacial contact, reduces the energy barrier for vapor nucleation, and achieves rapid boiling phase transition at ultra-low superheat, which effectively alleviates the delayed boiling initiation of traditional heat transfer surfaces at low temperatures.

By constructing copper-foam-based superhydrophobic nanofiber composite surfaces, this study systematically explores the coupling mechanism between micro/nano surface morphology, surface wettability, and key boiling heat transfer parameters, including boiling inception superheat, the HTC, and CHF. Most previous studies mainly focus on macroscopic boiling performance improvement, while this work establishes a multi-scale correlation between structural characteristics, interfacial properties, and thermal performance parameters, supplementing the mechanistic understanding of nanofiber-modified porous boiling surfaces. This work provides a feasible technical route for the design and preparation of high-efficiency low-temperature boiling heat transfer interfaces, and delivers a theoretical basis and technical reference for the performance optimization of low-temperature heat dissipation systems for high-heat-flux thermal equipment.

## 2. Materials and Methods

### 2.1. Materials

Polyvinylpyrrolidone (purity > 99%, Shanghai Aladdin Biochemical Technology Co., Ltd., Shanghai, China); tetraethyl orthosilicate (purity > 95%, Shanghai Aladdin Biochemical Technology Co., Ltd., Shanghai, China); perfluorodecyltrimethoxysilane; ethanol (≥99.8%); deionized water (self-prepared).

### 2.2. Sample Preparation and Characterization

The copper foam used for testing features a pore size of 60 μm and a porosity of 90%. High-temperature hydrogen reduction was performed in a vacuum sintering furnace to remove oxides and obtain clean copper foam. The target superhydrophobic SiO_2_ nanofibers were prepared by electrospinning and then modified to achieve superhydrophobicity.

**Figure 1:**
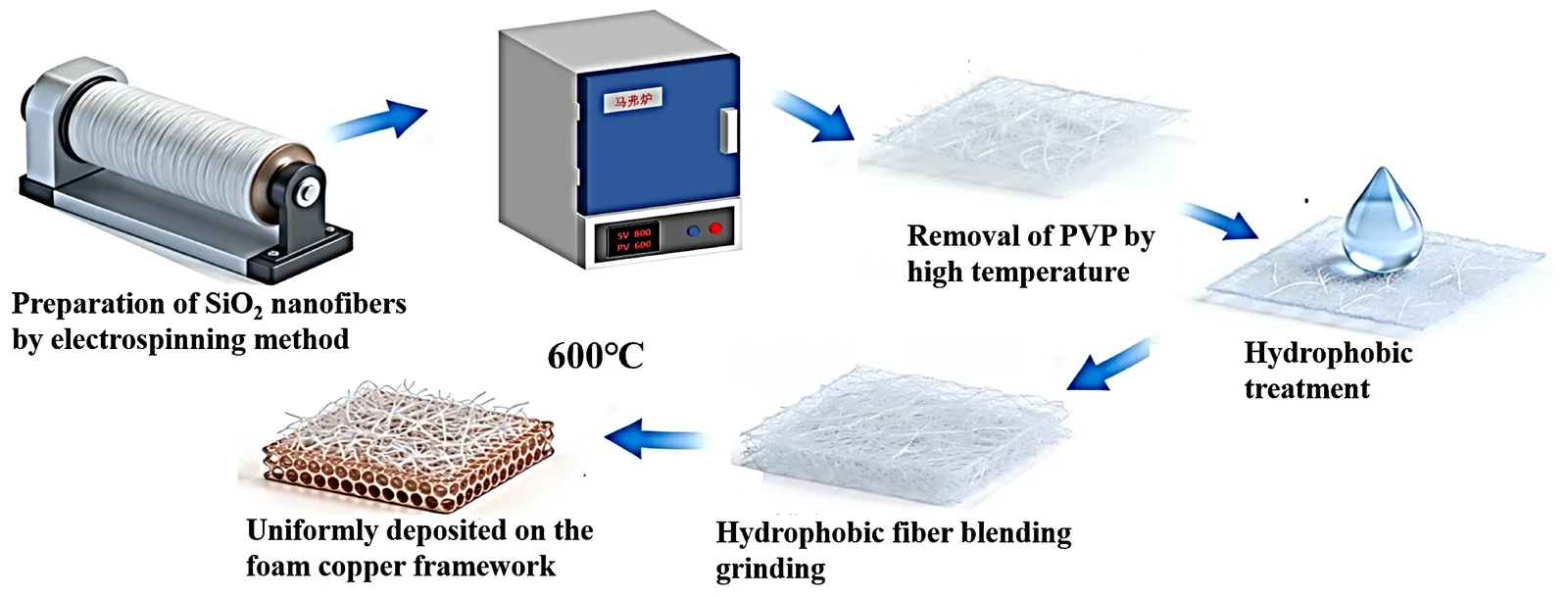
Process flux for the preparation of HCFs composite structure heat transfer surfaces.

Figure 1 depicts the workflow for constructing heat transfer surfaces with superhydrophobic micro/nanocomposite structures. To begin with, superhydrophobic SiO_2_ nanofibers were synthesized. Perfluorodecyltrimethoxysilane (1 mL) was mixed with water and ethanol, stirred for 12 h to complete hydrolysis, and aged for another 10 h. The SiO_2_ nanofibers pre-sintered at 600 °C in air were soaked in the as-prepared solution for 3 h, followed by manual grinding. The treated nanofibers were ultrasonically dispersed in absolute ethanol to form suspensions with varied concentrations. Copper foams were then treated by hydrothermal deposition to acquire the target micro/nanocomposite structures (Table 1).

### 2.3. Sample Characterization

Scanning electron microscopy (SEM), X-ray diffraction (XRD), and optical contact angle (OCA) measurements were adopted to characterize the micromorphology, chemical composition, and surface wettability of the as-prepared HCFs micro/nanocomposite structures, realizing a comprehensive multi-dimensional analysis.

**Figure 2:**
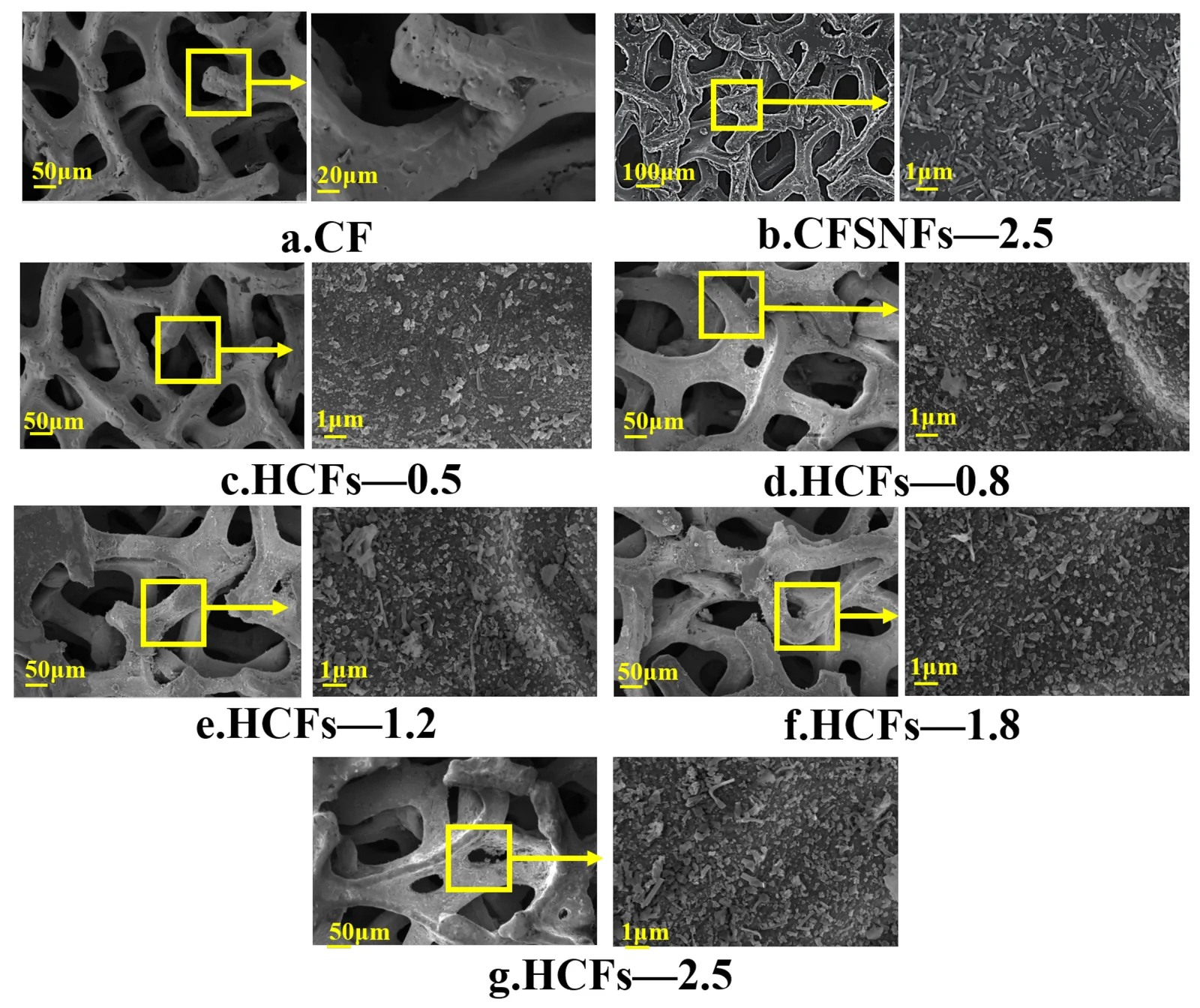
SEM analyses of HCFs modified surfaces, the yellow box denotes the magnified area, with the arrow indicating its enlarged view in the following figure.

Surface morphology and structure greatly influence heat transfer performance. SEM observations provide information on surface roughness, particle distribution, and porosity, which reflect the microstructure of materials. Figure 2 presents the SEM images of the superhydrophobic micro/nanocomposite structures.

As shown in the microscopic morphologies in Figure 2, pristine copper foam exhibits a regular and uniform porous structure. It features well-distributed open pores and an interpenetrating interior, where interconnected pore channels form a robust, continuous, three-dimensional network. This characteristic porous structure offers a large specific surface area and abundant anchoring sites for functional nanofillers, establishing a sound structural basis for composite fabrication.

For the composite samples in Figure 2b–f, their microstructures present an evident gradient variation. The loading amount of superhydrophobic SiO_2_ nanofibers increases gradually and uniformly from sample b–f, achieving controllable regulation of filler content in the composites. Microscopic characterization confirms that the as-prepared SiO_2_ nanofibers are densely and homogeneously distributed on the surface and inner pore walls of copper foam, with no obvious agglomeration, accumulation, or structural defects.

Furthermore, the nanofillers retain stable dimensions during gradient loading. The diameter of the superhydrophobic SiO_2_ nanofibers stays nearly identical across all samples, without obvious fluctuations. The stable morphology and uniform dispersion of the nanofibers demonstrate that the loading process does not damage the intrinsic structure of the nanofibers. Meanwhile, the porous copper foam can effectively immobilize and support the SiO_2_ nanofibers, which guarantees stable functional performance of the final composites.

**Figure 3:**
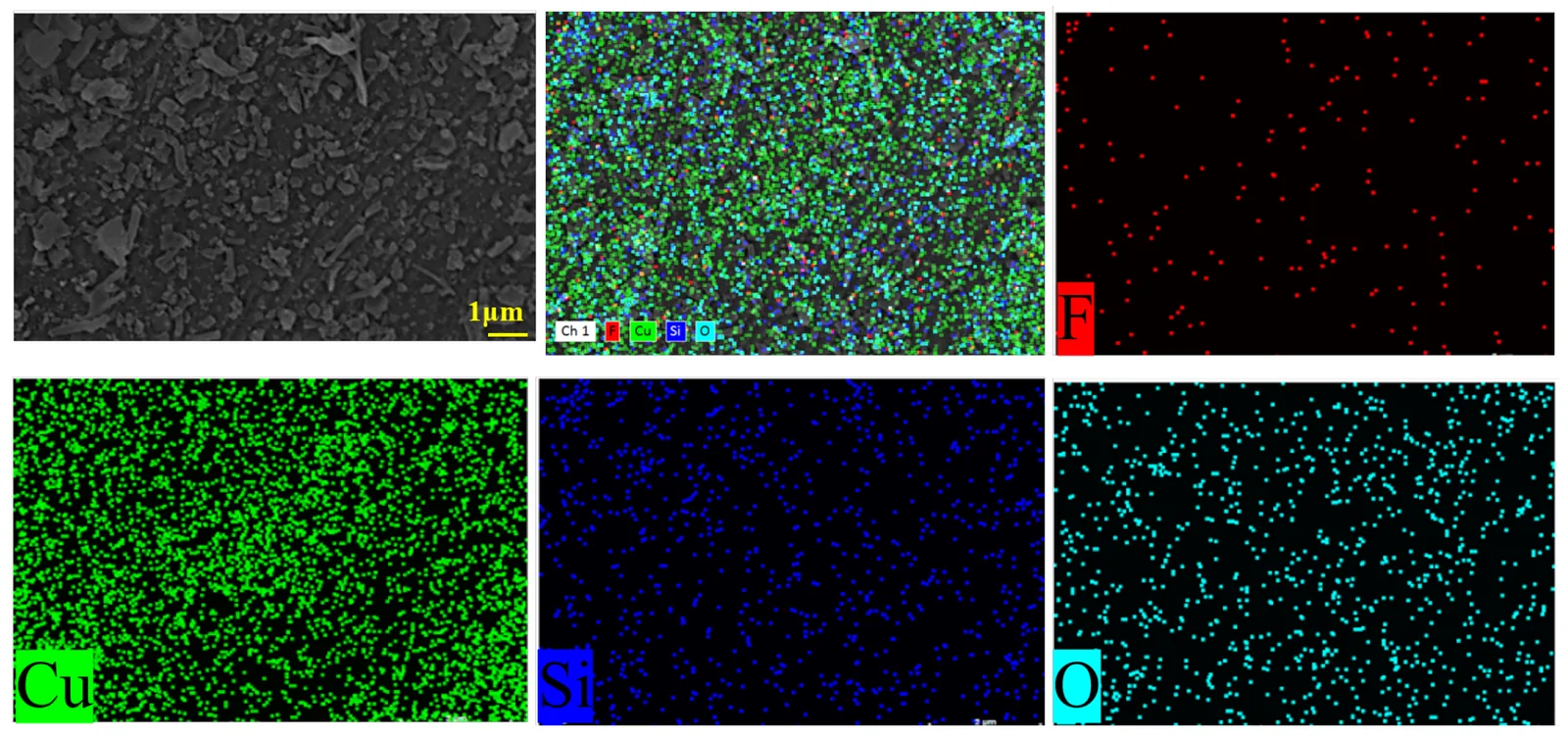
EDS test of the heat transfer HCFs surface.

EDS elemental mapping was performed to characterize the surface chemical composition and elemental distribution of the samples. The surface elemental composition and distribution exert an indirect influence on interfacial wettability, and thereby substantially affect the pool boiling heat transfer performance. As presented in Figure 3, four characteristic elements including copper, silicon, oxygen, and fluorine were detected on the surface of samples with micro/nanocomposite structures composed of superhydrophobic SiO_2_ nanofibers. Specifically, copper originates from the copper foam substrate, while silicon and oxygen are derived from the loaded SiO_2_ nanofibers. Fluorine, a typical low-surface-energy element, comes from the superhydrophobic coating after surface modification. The coexistence of these elements verifies the successful loading of SiO_2_ nanofibers on copper foam, as well as the effective implementation of superhydrophobic modification. This lays a solid material foundation for the formation of unique surface wettability and the achievement of superior pool boiling heat transfer performance.

**Figure 4:**
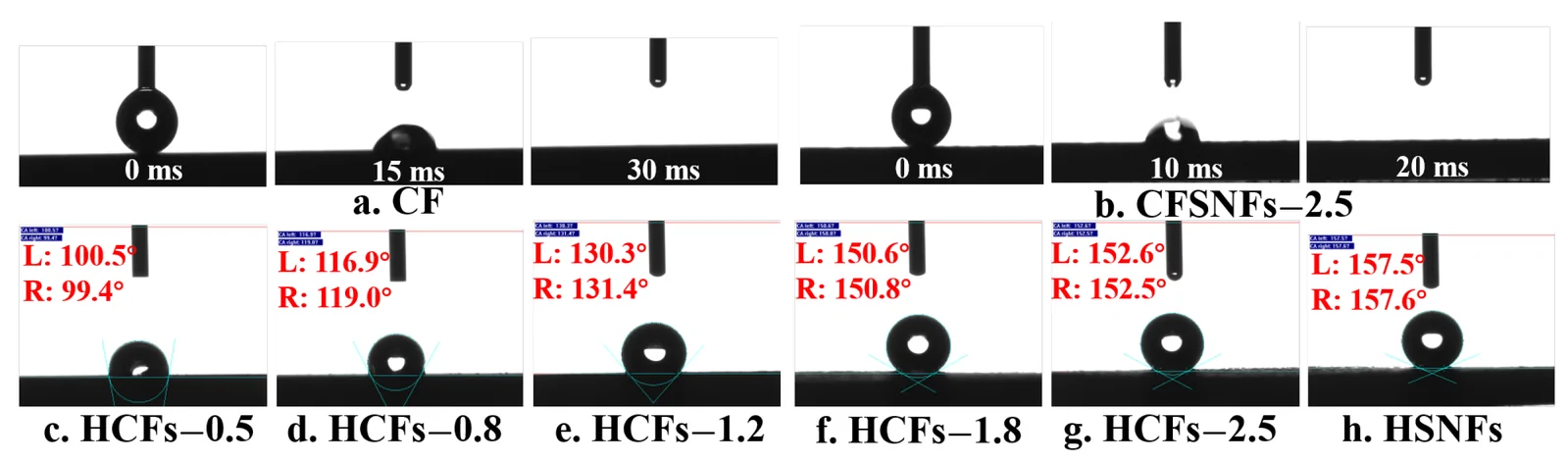
Contact angles of HCFs modified surfaces.

Contact angle characterization serves as a standard approach to quantify the effect of superhydrophobic surface treatment. According to the definition, a surface is hydrophobic at contact angles over 90°, and superhydrophobic once the contact angle surpasses 150°. The wettability of micro/nanocomposite structures with different deposition contents of superhydrophobic HCFs was measured using an OCA 20 contact angle goniometer, and the relevant data are displayed in Figure 4.

Figure 4 presents the wettability test results of micro/nanocomposite structures with different deposition amounts of SiO_2_ nanofibers on copper foam substrates. As a control group, the wettability characteristics of pristine untreated copper foam were first investigated. It was observed that a 0.05 mL water droplet could completely infiltrate into the surface of raw copper foam within 30 ms. This rapid water penetration behavior verifies that the original copper foam possesses excellent hydrophilic permeability, which is attributed to its interconnected three-dimensional porous network structure and intrinsic hydrophilic surface property. In contrast, the SiO_2_ nanofiber-modified surface fabricated via hydrophobic treatment exhibits a superior water-repellent performance, with a static contact angle reaching 157.5°, which meets the critical standard for superhydrophobic surfaces. For the prepared HCFs composite samples, their surface contact angles range from 100.5° to 152.6°. Notably, with the gradual increase in the deposition amount of superhydrophobic SiO_2_ nanofibers, the surface contact angle of HCFs increases monotonically and presents a stable upward trend. This variation law indicates that the loading content of superhydrophobic SiO_2_ nanofibers is the key factor regulating the surface wettability of composite materials. The increased deposition of nanofibers further optimizes the roughness of the micro/nanostructures and low-surface-energy characteristics of the sample surface, effectively inhibiting water droplet infiltration and significantly improving the hydrophobic performance of the composite surface.

**Figure 5:**
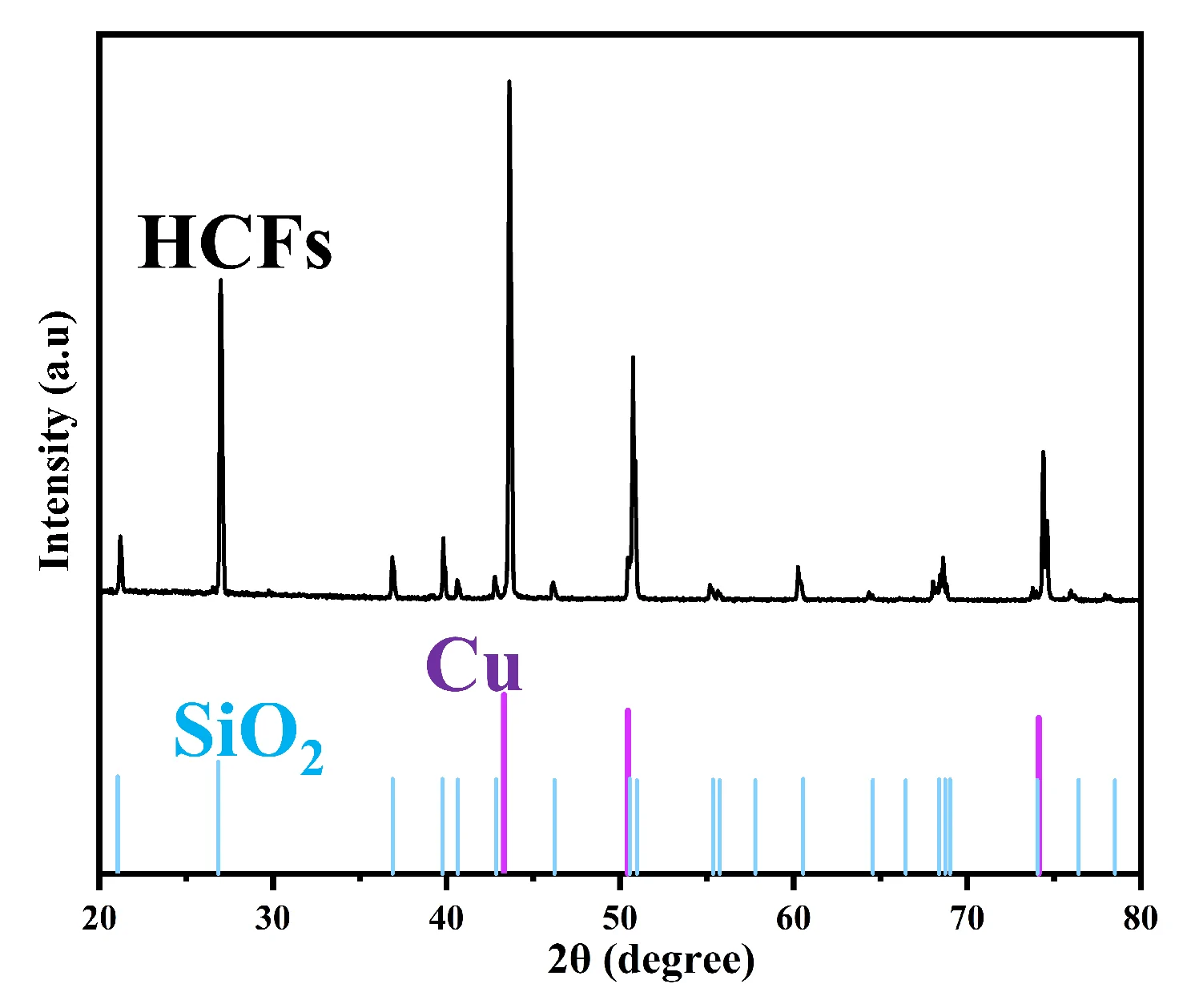
XRD spectra of HCFs composite structures.

The elemental and phase composition of heat transfer surfaces serve as the intrinsic factors governing the thermal performance of materials. Differences in crystal structure and composition directly alter the surface thermal resistance and thermal conductivity, thereby determining the overall heat transfer efficiency. To characterize the microscopic phase composition of the prepared heat transfer samples and clarify the influence of material composition on thermal performance, X-ray diffraction (XRD) tests were conducted. The corresponding XRD pattern of the sample is presented in Figure 5.

The characterization results clearly demonstrate that after reduction under a hydrogen atmosphere, the composite system consists solely of Cu and SiO_2_, with no other components detected. This indicates that no impurities were introduced during the reduction process, and the sample features a pure structure and uniform composition. Such a high-purity sample can effectively minimize experimental errors caused by interfering substances. With excellent stability and reliability, it fully meets the requirements for subsequent experimental investigations in this study.

### 2.4. Experimental Setup

**Figure 6:**
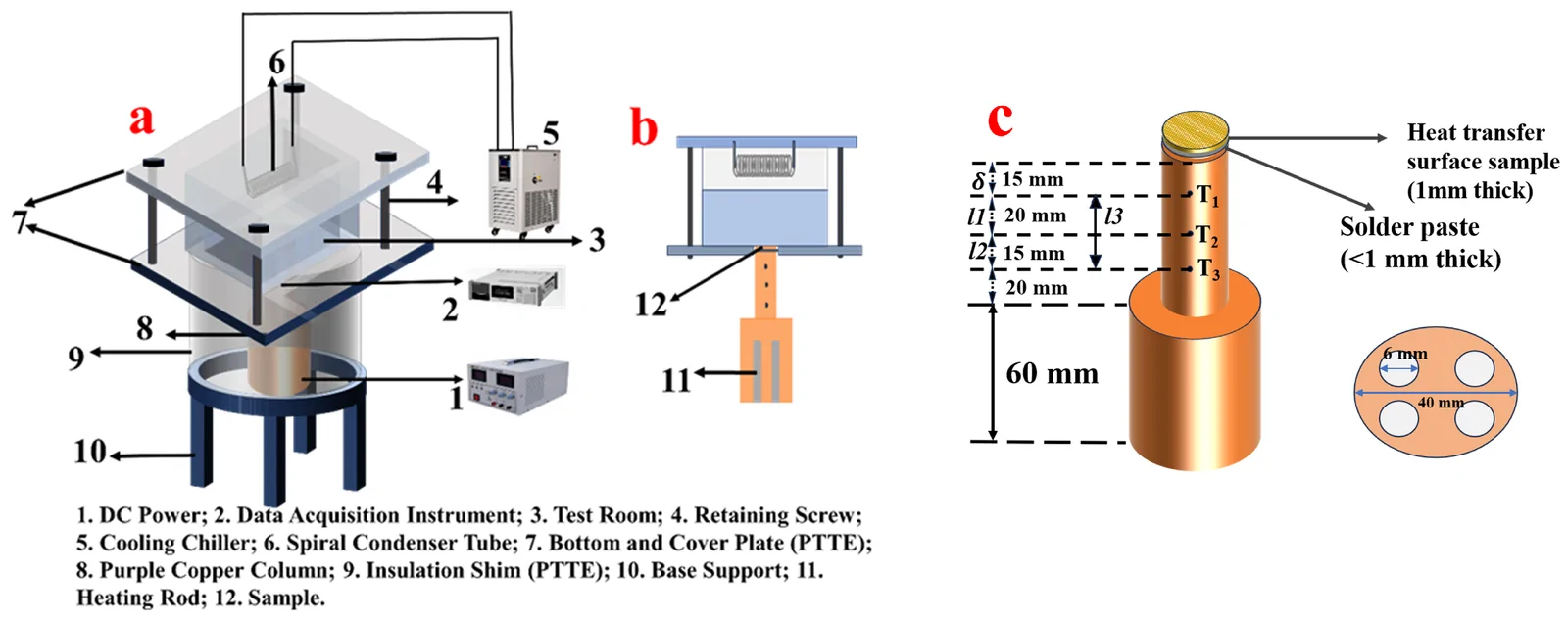
The pool boiling test setup. (**a**) Stereogram of the test platform. (**b**) Plan diagram of the test chamber. (**c**) Model diagram of heat-conducting copper column.

Pool boiling experiments were performed to evaluate the heat transfer performance of the fabricated HCFs micro/nanocomposite structures using the custom-built test platform illustrated in Figure 6. The experimental setup consists of a supporting framework, heating module, boiling chamber, data acquisition subsystem, and condensation reflux unit.

(1) The main support structure is fabricated from welded stainless steel to provide robust structural support for all other components.
(2) The heating system consists of two sections: the heat transfer section and the test specimen section. Thermal energy is provided by four high-precision single-ended cylindrical heating rods powered by DC power. As a core component, the purple copper column features three sidewall holes for temperature measurement using T-type thermocouples (Figure 6c). All external experimental parts are insulated with PTFE and 50 mm-thick quartz wool to minimize heat loss and ensure uniform heat distribution.
(3) The test room is assembled from a PTFE bottom plate, a PTFE cover plate, and a square quartz cylinder. Sealant is applied to seal the gap between the bottom plate and the quartz cylinder, while silicone sealant is used to seal the interface between the sample and bottom plate. A thermocouple is inserted through a reserved opening in the upper cover plate to measure the temperature of the working fluid inside the test room.
(4) The temperature acquisition system comprises three thermocouples mounted on the purple copper column and one thermocouple installed inside the test room. Temperature data is recorded using an Agilent data acquisition instrument, which is known for its stable performance and fast data transmission. The thermocouples are connected via T-type terminals, and real-time temperature data are continuously logged throughout the experiment.
(5) The condensation reflux system consists of a spiral condenser tube and a circulating coolant pump. The inlet and outlet of the spiral condenser tube are arranged on the cover plate and connected to the pump. This system regulates the condensation temperature, maintains atmospheric pressure in the boiling pool, and ensures a stable liquid level in the test room during the boiling process.

### 2.5. Computing Method

The Fourier steady-state differential equation for the heat transfer coefficient is used to determine the heat flux density and surface temperature of the heat transfer surface. All temperature measurements were recorded using type T thermocouples. During testing, real-time voltage (U) and current (I) are directly acquired from the displayed readouts of the pool boiling experimental setup (Figure 1) to reflect the actual heating input. A is defined as the circular contact surface of the test sample with a fixed radius of 1 mm, and this constant calculated area is used for all calculations. The heat flux density (q) of the heat-conducting column is calculated using Equation (1):

(1) $$q=\frac{U\times I\times 1000}{A},$$

where q is the heat flux, kW/m^2^; U is the voltage, V; I is the current, A; and A is the heat transfer area, m^2^.

The thermocouple-measured temperature can be used to compute the T_W_:

(2) $$T_{W}=T_{ave}-q\left(\frac{l_{s}}{k_{s}}+\frac{l_{c}}{k_{c}}\right),$$

where T_ave_ is the heat-conductivity column’s surface temperature, l_s_ and l_c_ are the thicknesses of the solder paste and test sample, respectively, and k_s_ and k_c_ represent the coefficients of the heat transfer coefficient of the solder paste and test sample, respectively. All thickness and thermal conductivity parameters are fixed constants calibrated by experimental settings. T_ave_ is calculated by Equation (3):

(3) $$T_{ave}=T_{1}-\frac{\delta }{3}\left(\frac{T_{2}-T_{1}}{l1}+\frac{T_{3}-T_{2}}{l2}+\frac{T_{3}-T_{1}}{l3}\right),$$

where δ is the distance from the upper measuring point of the heat-conducting copper column to the column top, and l denotes the distance between thermocouples, as illustrated in Figure 6c.

Wall superheat (ΔT) is calculated by subtracting the heat transfer surface temperature from the working fluid surface temperature.

(4) $$\Delta T=T_{W}-T_{sat},$$

where T_sat_ is the fluid saturation temperature.

The final calculation of the boiling heat transfer coefficient (h) is expressed as:

(5) $$h=\frac{q}{∆T}.$$

### 2.6. Error Analysis

During the pool boiling heat transfer test, various influencing factors will alter the heat transfer performance and bring about experimental errors. To ensure the validity of the final results, we carry out a detailed analysis on error sources. The experimental errors are mainly grouped into systematic errors and measurement errors. Specifically, systematic errors are associated with the heat transfer surface area, machining accuracy of the heat-conducting copper column, and thermocouple placement. Measurement errors are derived from the precision of the DC power supply, data acquisition system, thermocouples, and heating resistance.

The heat-conducting copper columns and graphite molds both have a machining tolerance of ±0.01 mm. Double-layer PTFE and quartz wool insulation was utilized to reduce heat loss. PTFE has a thermal conductivity of 0.24 W·m^−1^·K^−1^, and quartz wool ranges from 0.15 to 0.37 W·m^−1^·K^−1^. The Fourier heat conduction formula indicates that the experimental setup has a heat loss below 2.2%.

Measurement errors stem from data acquisition, DC power output, thermocouples, and heating resistance. The relevant errors are 1.2% (DC power output), 0.15 K (thermocouple measurement), 1.2% (thermal conductivity), and 1.4% (effective heat dissipation area of the heat transfer surface).

Experimental measurements are adopted to acquire these parameters, so uncertainty evaluation is indispensable. Based on the error propagation analysis method proposed by Kline [32] and McClintock [33], where δ represents the standard error, assume the indirectly measured quantity f and the directly measured quantities x_1_, x_2_, …, x_m_ follow the functional relationship f = f (x_1_, x_2_, …, x_m_), with x_1_, x_2_, …, x_m_ being uncorrelated variables. In this case, the standard error of f conforms to the following propagation equation:

(6) $${\delta^{2}}_{f}=\sum_{i=1}^{m}{\left(\frac{\partial F}{{\partial x}_{i}}\right)}^{2}{\delta_{i}}^{2},$$

(7) $$\frac{\delta q}{q}=\sqrt{{\left(\frac{{\delta T}_{1}}{{\Delta T}_{13}}\right)}^{2}+{\left(\frac{{\delta T}_{3}}{{\Delta T}_{13}}\right)}^{2}+{\left(\frac{{\delta L}_{13}}{{\Delta L}_{13}}\right)}^{2}},$$

(8) $$\frac{\delta h}{h}=\sqrt{{\left(\frac{\delta q}{q}\right)}^{2}+{\left(\frac{{\delta T}_{3}}{T_{s}-T_{sat}}\right)}^{2}+{\left(\frac{{\delta T}_{sat}}{T_{s}-T_{sat}}\right)}^{2}},$$

and

(9) $$\frac{\delta T}{T}=\sqrt{{\left(\frac{{\delta T}_{1}}{T_{s}}\right)}^{2}+{\left(\frac{{Ls}_{1}}{{kC}_{u}}\cdot \frac{\delta q}{T_{s}}\right)}^{2}+{\left(\frac{q}{{kC}_{u}}\cdot \frac{{\delta Ls}_{1}}{T_{s}}\right)}^{2}+{\left(\frac{{qLs}_{1}}{{k^{2}C}_{u}}\cdot \frac{\delta {kC}_{u}}{T_{s}}\right)}^{2}},$$

where $\delta {kC}_{u}$ is the uncertainty of copper column thermal conductivity, equal to roughly 1%. Heat flux density has an uncertainty of 5.4%, and the uncertainties of the pool boiling heat transfer coefficient and surface temperature are 7.4% and 6.6%.

When CHF occurs on the P surface of the boiling device, the uncertainties of q, ΔT, and Δh are 4.36%, 5.78%, and 6.82%.

## 3. Experimental Process

**Figure 7:**
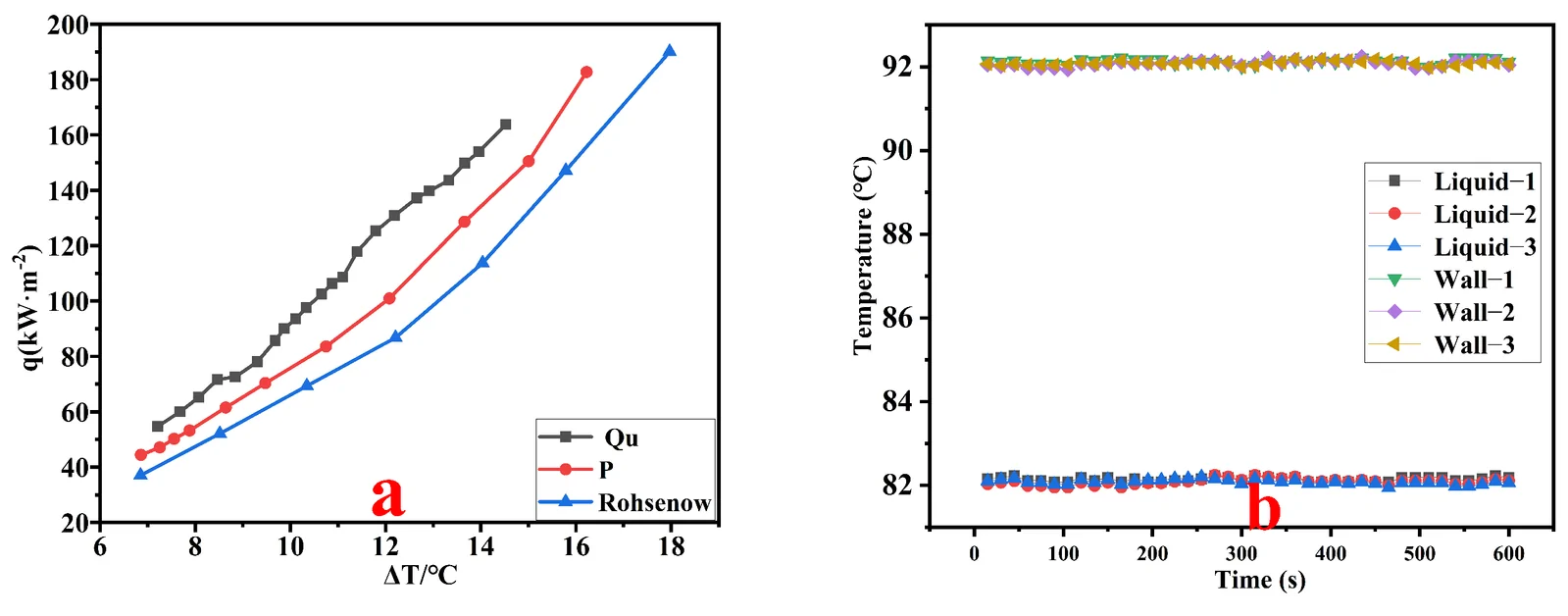
Reliability of the test system. (**a**) Boiling curve in uncoated pool boiling; (**b**) temperature measurements.

Figure 7 presents the reliability verification of boiling curves. Figure 7a shows that the boiling curves from Qu [34] and Rohsenow [35] agree well with the current data, confirming the validity of our experimental results. Figure 7b plots the surface and liquid temperature variations with time for deionized water boiled on the P surface. Three repeated measurements were performed under the same heating conditions. The average difference between the surface temperature and fluid temperature is within 0.2 °C, which proves stable pool boiling performance and reliable experimental facilities.

## 4. Results and Discussion

### 4.1. Pool Boiling Heat Transfer Performance Testing and Analysis

**Figure 8:**
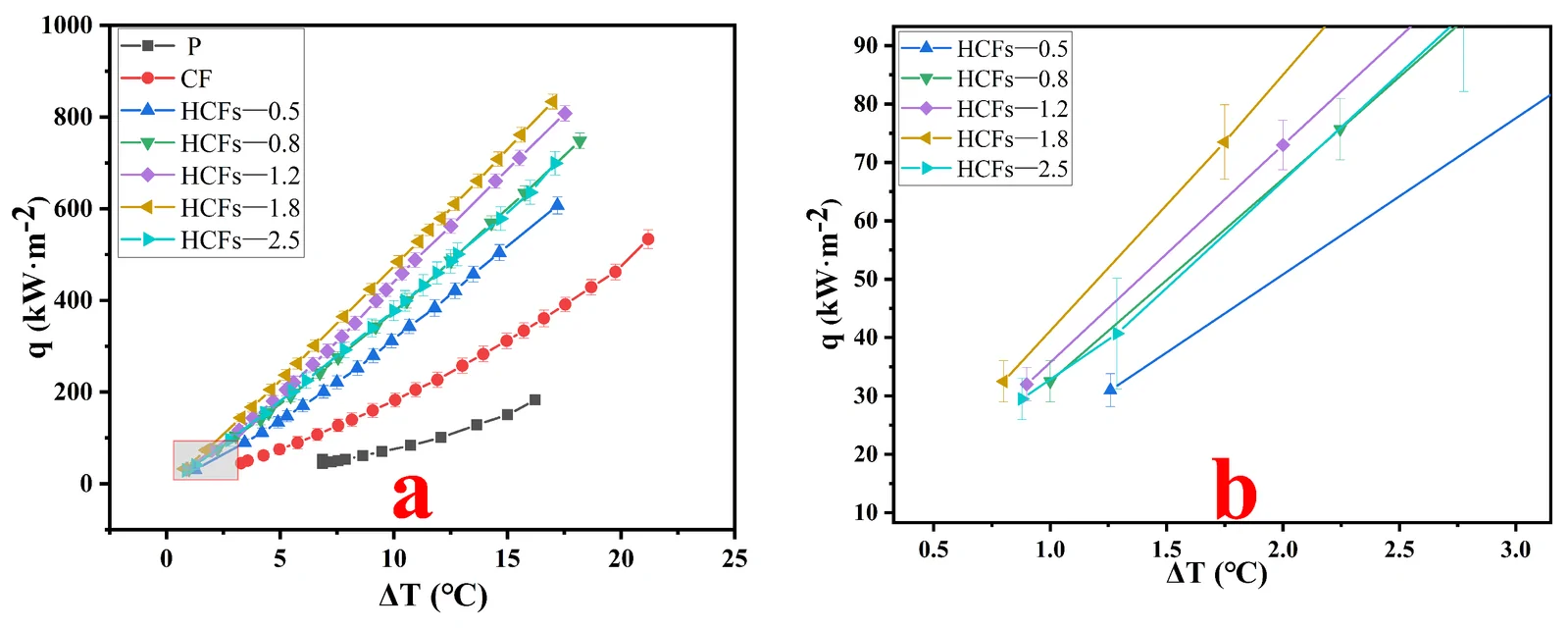
Heat transfer performance test of HCFs. (**a**) The variation curve of q with ΔT; (**b**) local magnified view of the overlapping region in (**a**).

Deionized water without non-condensable vapor was used to test the pool boiling performance of polished surfaces, copper foam, and HCFs micro/nanocomposites. The HCFs were prepared via hydrothermal deposition of superhydrophobic SiO_2_ nanofibers on copper foam. After material characterization, boiling curves in Figure 8 were acquired to analyze the influence of nanofibers on boiling behaviors.

Figure 8 compares the heat flux versus wall superheat characteristics of smooth copper, pure copper foam (CF), and superhydrophobic SiO_2_ nanofiber-modified copper foam (HCFs). The enhanced pool boiling performance of HCFs stems from the synergistic coupling of a three-dimensional porous skeleton and uniform nanofiber interfacial modification. Unlike conventional smooth hydrophobic surfaces that readily form continuous insulating vapor films and deteriorate critical heat flux (CHF), the interconnected porous architecture provides stable capillary liquid replenishment and suppresses lateral vapor propagation. Therefore, the overall boiling performance is governed by the trade-off between nanofiber-facilitated bubble nucleation and porous-media-based two-phase transport, rather than intrinsic surface hydrophobicity alone.

During boiling incipience, smooth copper exhibits a high nucleation barrier with an onset nucleate boiling (ONB) superheat of 6.8 °C due to its high surface free energy and limited geometric nucleation cavities. In contrast, superhydrophobic SiO_2_ nanofiber modification reduces interfacial free energy and nucleation resistance, while the hierarchical porous copper framework provides abundant and stable active nucleation sites. Increased nanofiber loading homogenizes surface hydrophobic modification and progressively lowers ONB superheat. Both HCFs—1.8 and HCFs—2.5 achieve an extremely low ONB superheat of 0.8 °C, greatly facilitating bubble incipience under mild heating conditions.

However, reduced ONB superheat cannot independently determine optimal comprehensive boiling performance. Pure copper foam and smooth copper deliver limited CHF values, while HCFs—1.8 achieves a prominently improved CHF of 845.12 kW·m^−2^. Benefiting from rationally tuned interfacial wettability and unobstructed porous channels, HCFs—1.8 effectively restrains large-scale vapor film formation and maintains stable boiling heat transfer. In comparison, excessive nanofiber loading causes porous channel blockage, elevates two-phase flow resistance, and hinders liquid reflux and vapor evacuation. The deteriorated interfacial two-phase transport induces local vapor accumulation, thereby degrading the boiling performance of overloaded HCFs samples.

**Figure 9:**
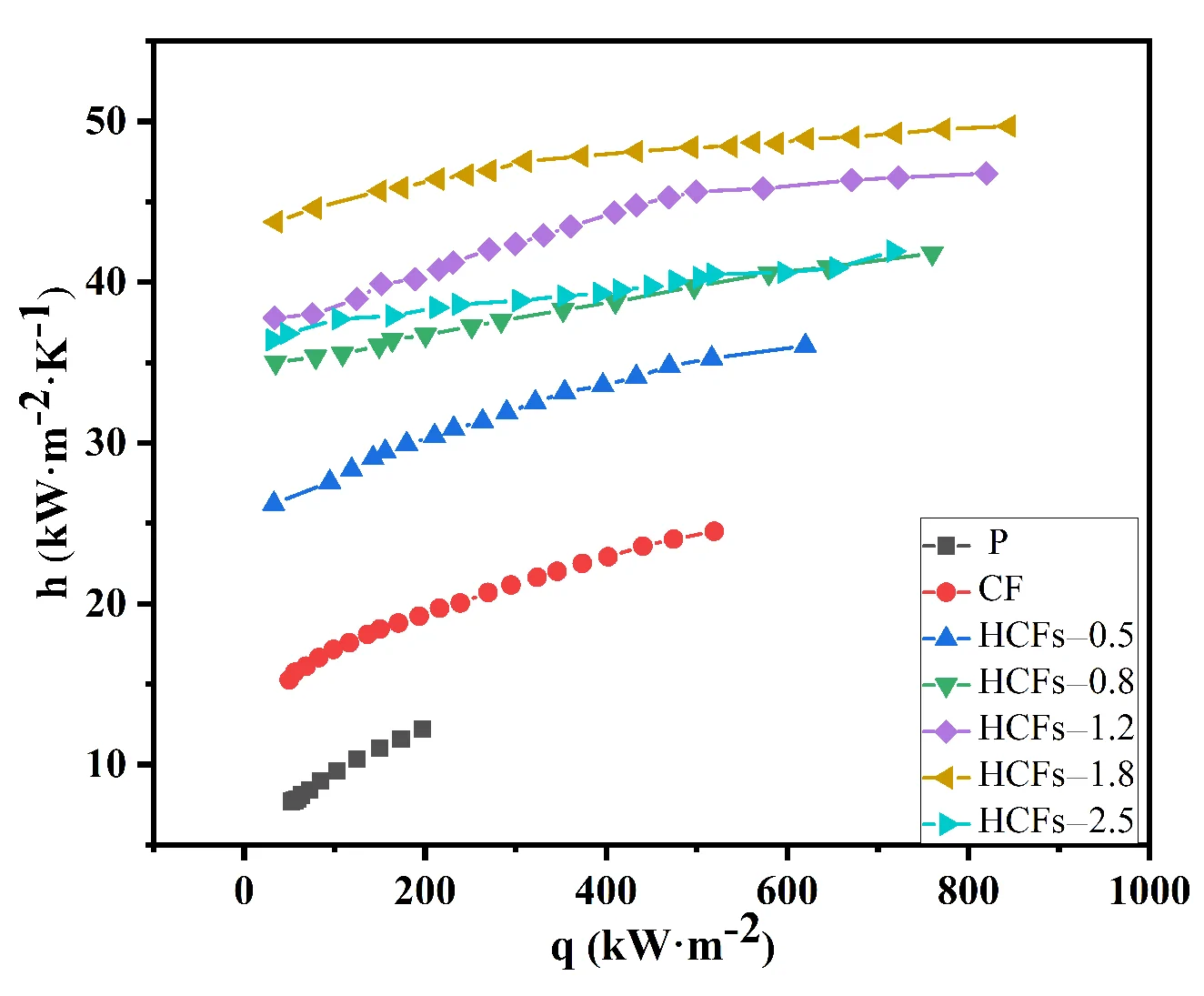
Heat transfer performance test of HCFs: the variation in the HTC with heat flux density.

Figure 9 further illustrates the heat transfer coefficient (HTC) evolution with heat flux. Smooth copper and pure copper foam present relatively low HTC values, while HCFs—1.8 exhibits the maximum HTC and the fastest HTC growth rate under high heat flux, demonstrating superior interfacial heat transfer capacity. The non-monotonic loading-dependent HTC trend is determined by the competitive balance between bubble nucleation enhancement and interfacial thermal resistance variation. Although SiO_2_ possesses a much lower intrinsic thermal conductivity than copper, moderately loaded nanofibers are uniformly and discretely distributed on the foam surface and internal pore walls. The high-conductivity copper skeleton dominates the major heat conduction pathway, while dispersed SiO_2_ micro/nanostructures act as high-efficiency nucleation cavities. The regulation of prominent bubble dynamics fully compensates for the minor thermal resistance penalty, enabling sustainable HTC improvement at nanofiber loadings below 1.8 mg.

Once the nanofiber loading exceeds 1.8 mg, excessive nanofibers uniformly cover the skeleton surface and densely fill internal porous channels. The thickened low-conductivity modification layer increases overall interfacial thermal resistance, while pore blockage impairs capillary-driven liquid replenishment and aggravates vapor retention. The coupled adverse effects of elevated thermal resistance and degraded two-phase transport offset the nucleation-promoting advantage, resulting in a deteriorated HTC for overloaded samples. Overall, the optimized HCFs—1.8 achieves a delicate balance between active nucleation, vapor evacuation, and liquid reflux, delivering excellent comprehensive pool boiling heat transfer performance.

**Figure 10:**
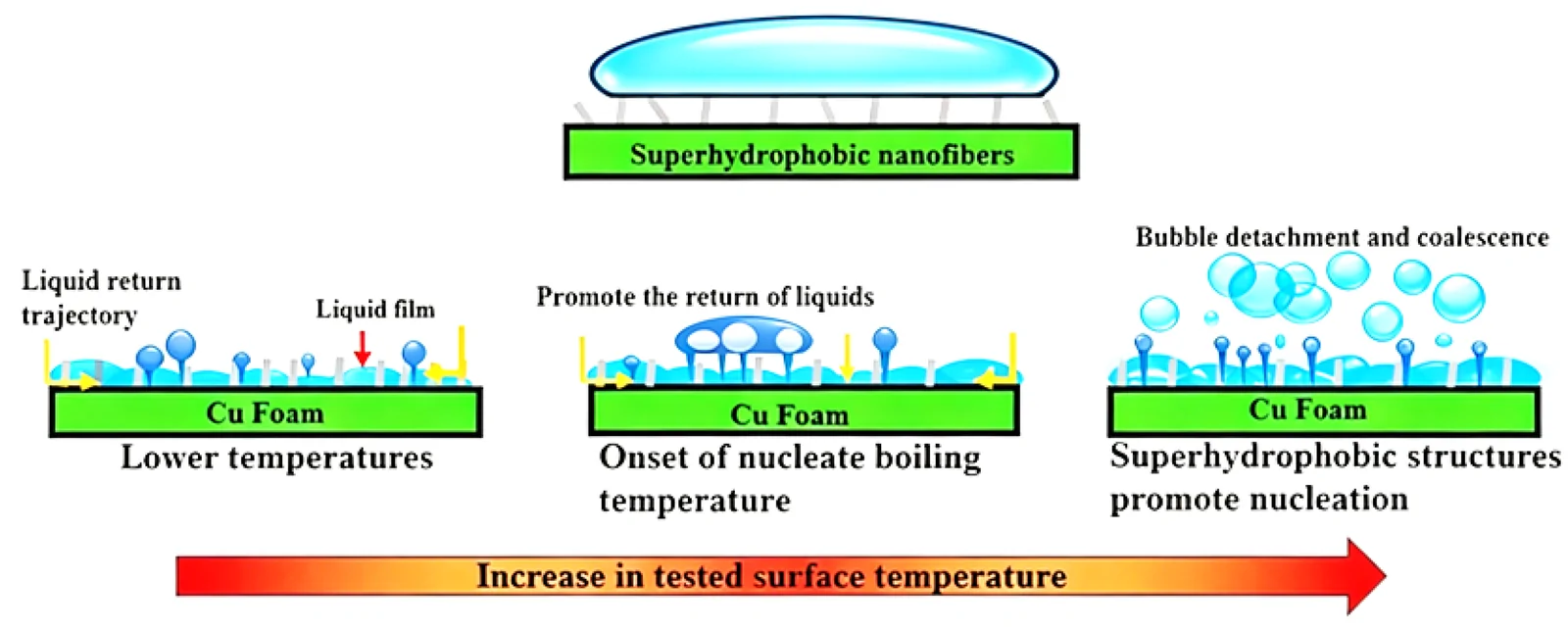
The mechanism of pool boiling with HCFs surface.

HCFs micro/nanocomposites integrate micro-scale porous frameworks with superhydrophobic nanofibers, and the corresponding mechanism diagram is shown in Figure 10. This composite structure expands the heat transfer specific surface area and increases the quantity of active nucleation sites. As a result, bubbles detach at low superheat and liquid reflows rapidly. The intrinsic superhydrophobic property further optimizes the overall heat transfer behavior.

For all tested surfaces, a reasonable bubble departure size and nucleation site density are vital to keep stable boiling performance. Under low heat flux, HCFs present prominent boiling advantages. The matching of superhydrophobic nanofibers and hydrophilic porous substrates effectively induces bubble generation and restrains bubble coalescence. Moreover, the residual cavities after bubble departure can act as new nucleation sites, sustaining continuous bubble growth and detachment throughout the boiling process.

### 4.2. Calculation of Bubble Dynamics Parameters

Jensen and Memmel [36] proposed a classical correlation for active nucleation site density based on systematic pool boiling experimental investigations. This correlation is adopted in the present work owing to its good applicability to heating surfaces with micro/nanocomposite structures.

(10) $$D_{d}=0.19{\left(1.8+10^{5}\frac{Ja}{Pr_{l}}{\left[\frac{\Delta \rho g}{\rho_{l}\nu_{l}^{2}}{\left(\frac{\sigma }{\Delta \rho g}\right)}^{2/3}\right]}^{-1}\right)}^{2/3}{\left(\frac{\sigma }{\Delta \rho g}\right)}^{1/2},$$

where $\rho_{l}$ represents the fluid density; ν_l_ denotes the kinematic viscosity, Ja is the Jakob number; and Pr is the Prandtl number.

Based on the Young–Laplace [37] and Clausius–Clapeyron equations, shrinking the bubble radius will raise the pressure and temperature inside the bubbles. Researchers adopt a linear model to describe the fluid temperature gradient in the wall boundary layer, and the liquid phase temperature reaches its minimum at the top of nucleating bubbles. On these grounds, Griffith [38] proposed the definition of the critical nucleation radius as:

(11) $$R_{c}=\frac{{2\sigma T}_{sat}}{\rho_{v}h_{lv}{∆T}_{sat}},$$

where $h_{lv}$ denotes the latent heat of evaporation, $\rho_{v}$ is the vapor density, and σ represents the surface tension.

Kocamustafaogullar [39] established the classical boiling theory, which is adopted in this work. The HCFs composite effectively increases nucleation point density (N_a_), decreases boiling wall superheat, and improves the boiling initiation phase. Nucleation point density is expressed as follows:

(12) $$N_{a}D_{d}^{2}=2.157\times 10^{-7}{\left(\frac{\rho_{\nu }}{\rho_{l}-\rho_{\nu }}\right)}^{3.2}{\left(1+0.0049\frac{\rho_{l}-\rho_{\nu }}{\rho_{\nu }}\right)}^{4.13}{\left(\frac{D_{d}}{2R_{c}}\right)}^{4.4}.$$

According to the analysis of Sakashita [40], the bubble detachment frequency (f) is given by:

(13) $$f=0.6{\left[\frac{g\left(\rho_{l}-\rho_{v}\right)}{\rho_{l}}\right]}^{\frac{2}{3}}{\left\{\nu_{l}{\left[\frac{g\left(\rho_{l}-\rho_{v}\right)\rho_{l}^{2}\nu_{l}^{4}}{\sigma^{3}}\right]}^{-0.25}\right\}}^{-\frac{1}{3}}.$$

As a key characteristic, the bubble detachment frequency is negatively correlated with the bubble detachment diameter. A larger detachment diameter corresponds to a lower frequency, and vice versa. Derivation of this frequency depends on bubble growth time and residence time, and nucleation exerts a dominant effect on bubble growth time.

**Figure 11:**
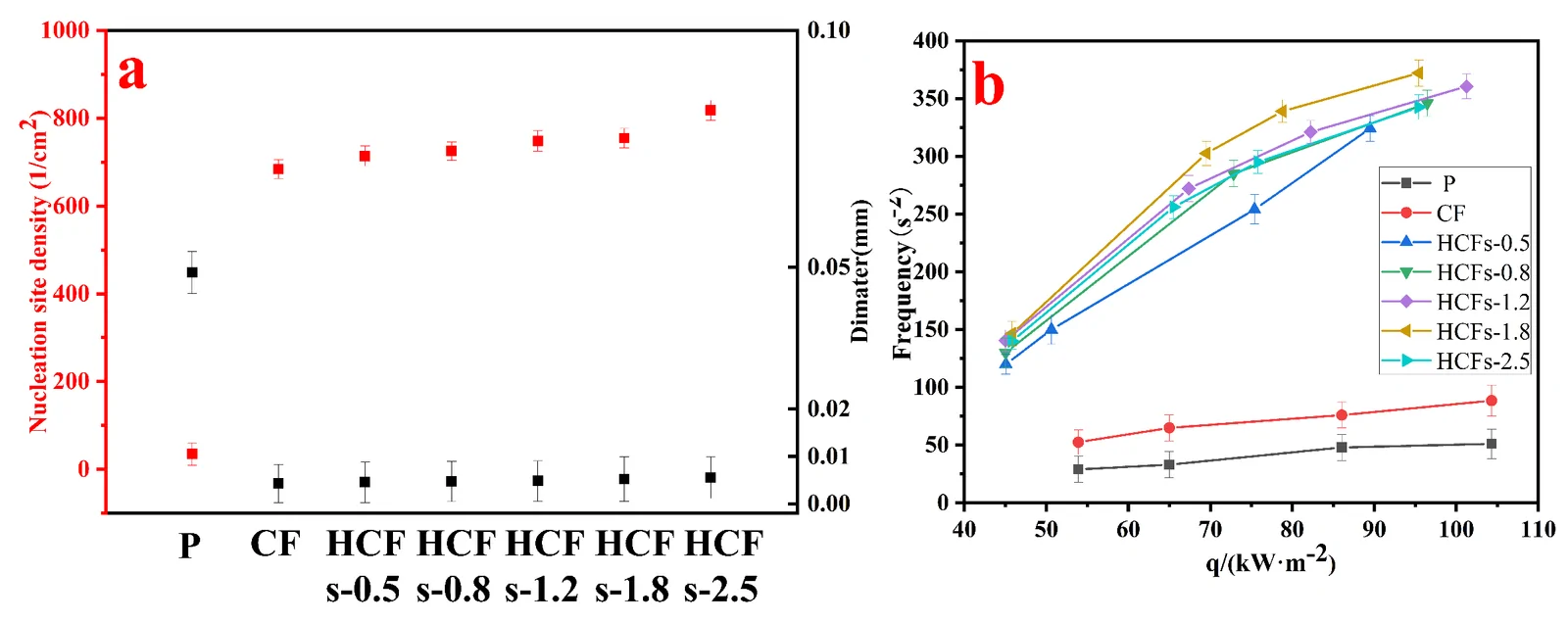
Study on bubble dynamic parameters (ONB). (**a**) Number of nucleation sites regarding density and diameter, and (**b**) frequency.

Figure 11a plots the calculated number of nucleation sites against bubble diameter based on the foregoing equations, and Figure 11b illustrates the bubble detachment frequency. Superhydrophobic structures exhibit superior wettability relative to hydrophilic ones, along with higher nucleation site density, a larger bubble detachment diameter, and lower detachment frequency. With the increased loading of superhydrophobic SiO_2_ nanofibers on copper foam, the surface nucleation sites and contact angle continuously grow, while the bubble detachment diameter increases and the detachment frequency declines.

It is concluded that the porous copper foam and superhydrophobic nanofibers introduce abundant nucleation sites for heat transfer surfaces. Copper foam offers a large specific surface area, and nanofibers contribute to more vaporization cores. Bubbles remain on superhydrophobic surfaces for a longer time and detach at a larger size. The synergistic effect of the porous structure and superhydrophobic SiO_2_ nanofibers raises the density of vaporization cores, which ultimately improves the pool boiling heat transfer capability.

Table 2 compares mainstream fabrication routes with the electrospinning-assisted fiber deposition strategy in this work. Conventional approaches are plagued by inadequate porous-structure tunability, microcrack propensity, and conductive-substrate prerequisites, which degrade long-term boiling stability. The present method fabricates robust interconnected nanofiber networks on copper foam, yielding simultaneous enhancements in CHF and the HTC. Notwithstanding calcination-induced cost overheads, this technique offers a feasible alternative for high-performance boiling-surface engineering with competitive thermal–hydraulic performance.

### 4.3. CFD Numerical Analysis of HCFs Composite Structures

The numerical investigations in this work were performed based on the Fluent module of the ANSYS Workbench 2023 R2 platform. Computational fluid dynamics (CFD) serves as a typical numerical computation method rooted in hydrodynamic theories. It realizes the prediction and analysis of fluid flow characteristics and corresponding physical processes through the discrete solution of the governing equations of fluid motion. The precision and reliability of numerical CFD simulations are determined by the rational simplification of practical physical models, together with the mathematical descriptions of three basic physical conservation principles, including mass conservation, momentum conservation, and energy conservation.

Mass equation:

(14) $$\frac{\partial \rho }{\partial t}+div\left(\rho U\right)=0.$$

Energy equations:

(15) $$\frac{\partial \left(\rho u\right)}{\partial t}+div\left(\rho uU\right)=div\left(\eta gradu\right)+S_{u}-\frac{\partial P}{\partial x}.$$

(16) $$\frac{\partial \left(\rho v\right)}{\partial t}+div\left(\rho vU\right)=div\left(\eta gradv\right)+S_{v}-\frac{\partial P}{\partial y}.$$

(17) $$\frac{\partial \left(\rho w\right)}{\partial t}+div\left(\rho wU\right)=div\left(\eta gradw\right)+S_{w}-\frac{\partial P}{\partial z}.$$

Momentum equation:

(18) $$\frac{\partial \left(\rho T\right)}{\partial t}+div\left(\rho UT\right)=div\left(\frac{\lambda }{c_{p}}gradT\right)+S_{T}.$$

**Figure 12:**
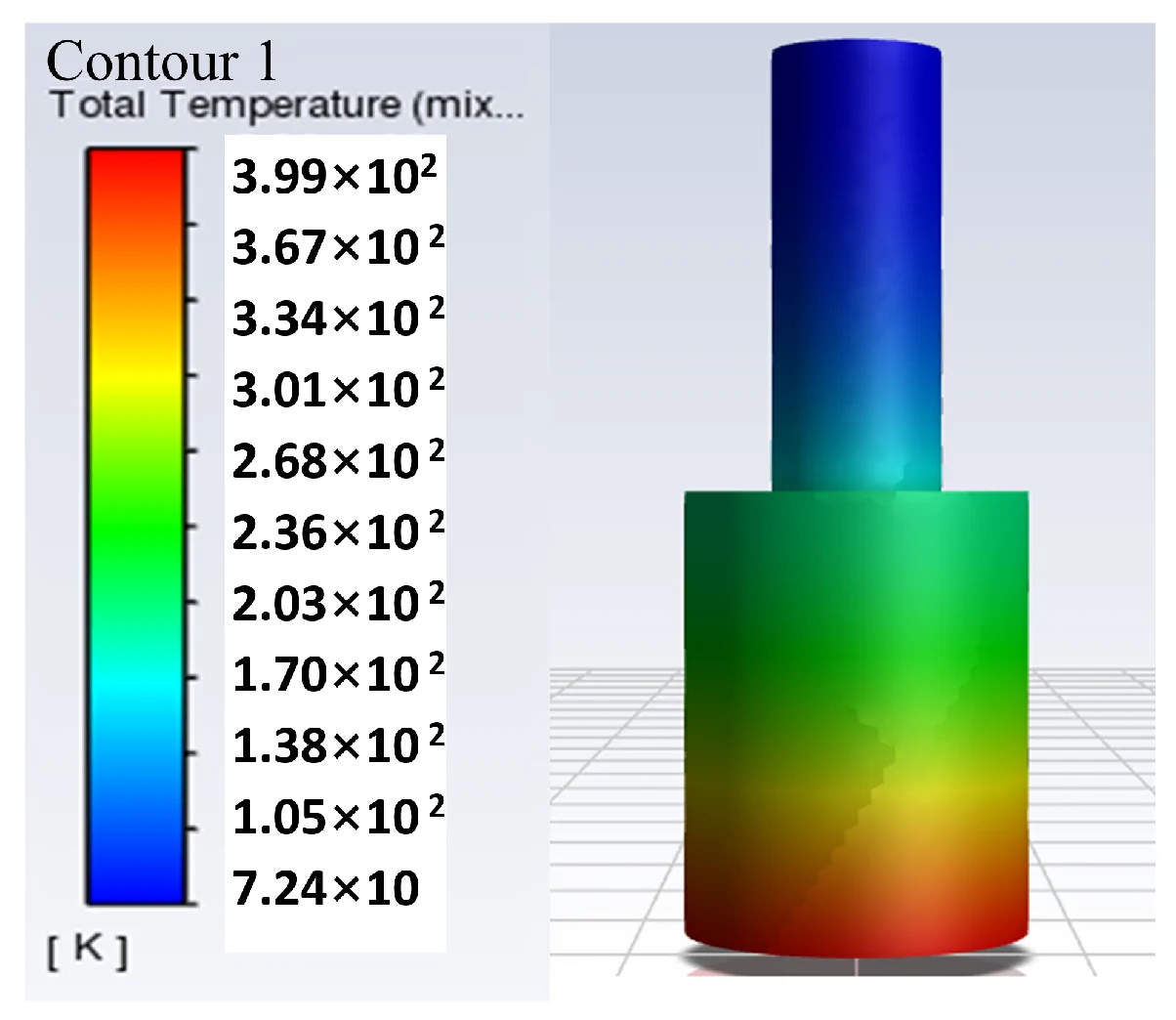
Heat distribution diagram of the heat-conducting copper column.

Numerical simulation relies fundamentally on model construction, where the precision of the physical model governs the accuracy of simulated results. Ansys Workbench was employed to characterize the heat transfer surface of HCFs composites. Specifically, DesignModeler was used for geometric modeling, Fluent Meshing for meshing, Fluent for numerical simulation, and CFD-Post for result post-processing. Before conducting simulations, the thermal conductivity of the copper column of the test specimen was tested, as shown in Figure 12.

**Figure 13:**
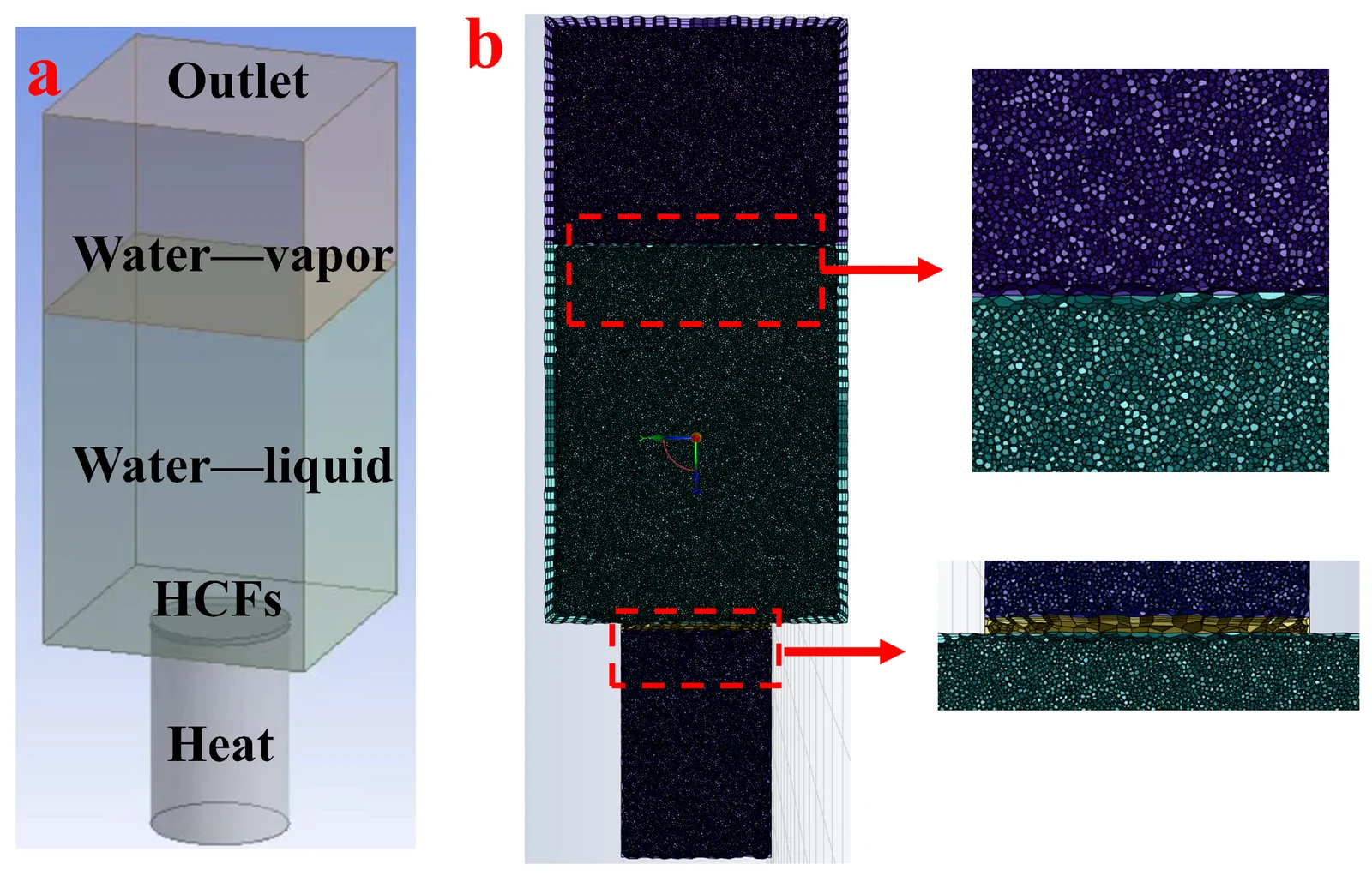
(**a**) The simplified model. (**b**) Mesh results of the model. The red box marks the magnified region, the arrow points to this area, and the magnified view is shown at the corresponding position.

CFD simulation generally involves five key steps: geometric modeling, meshing, boundary condition setup, numerical computation, and post-processing. Parametric geometric modeling was performed in DesignModeler according to the actual dimensions of test samples. The geometric model was reasonably simplified by removing trivial structures, including supports and thermal insulation facilities that exert negligible influences on computational accuracy, as illustrated in Figure 13a, with all model parameters calibrated to match practical working conditions. The established model was then imported into the Meshing module for computational domain discretization, as presented in Figure 13b.

**Figure 14:**
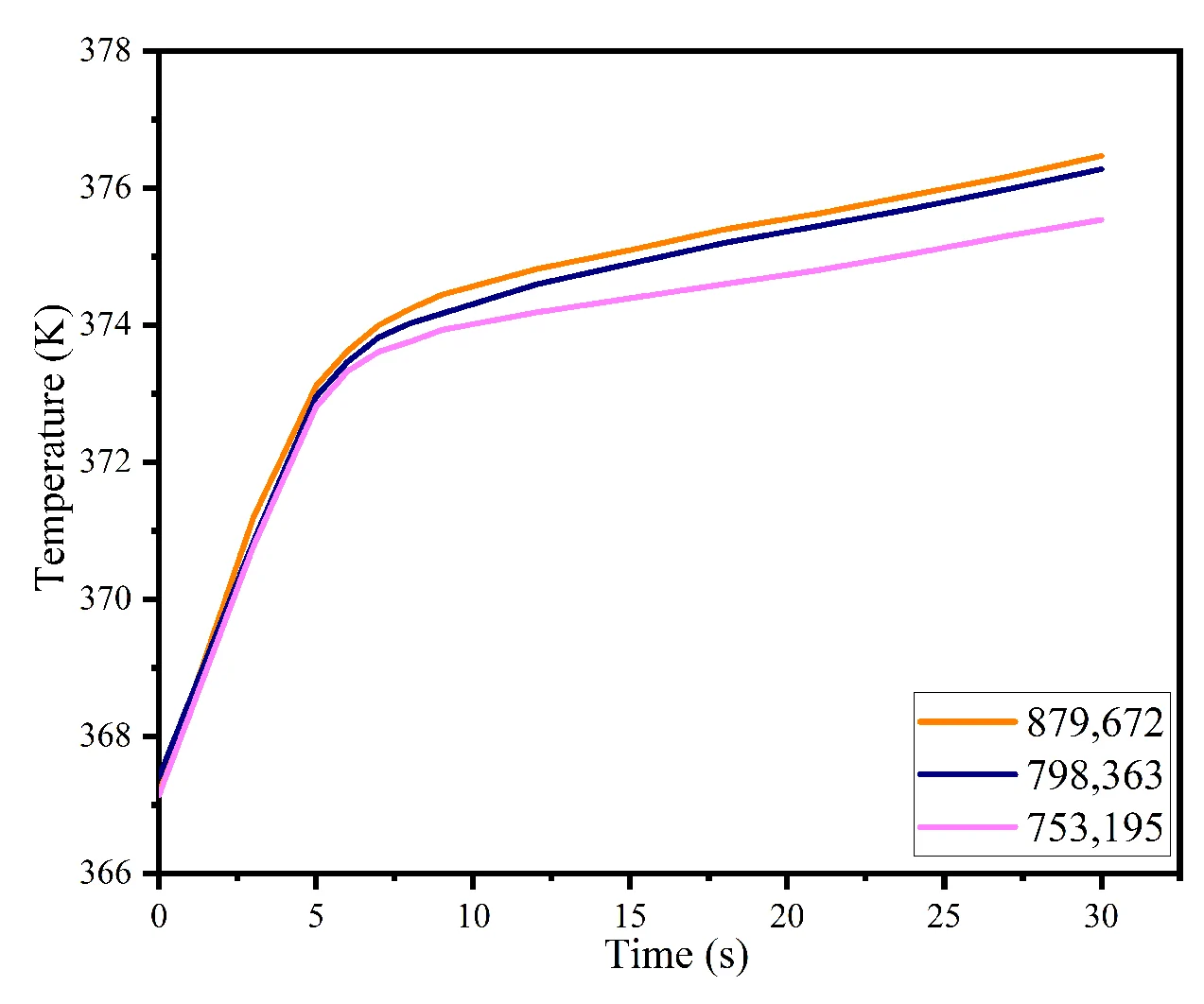
Mesh independence test.

The vertical boundaries of the solid–liquid domain were assigned adiabatic conditions. A constant heat flux was imposed on the bottom heating surface to deliver heat toward the solid–liquid interface under standard atmospheric pressure, while the top was defined as a pressure outlet. Mesh independence verification was conducted to optimize simulation accuracy. The total number of mesh cells in the final model was 843,764, as illustrated in Figure 14.

The energy equation was activated. The VOF model was employed to characterize dynamic gas–liquid interface behaviors and bubble morphology on hydrophobic surfaces. All materials were loaded and allocated to respective parts, where copper and SiO_2_ represented solid phases, water the liquid phase, and water vapor the gas phase. The contact angle was set as the boundary condition for hydrophobic surfaces to simulate droplet wetting characteristics. The gas–liquid interfacial tension was calculated according to Equation (19) [44].

(19) $$\sigma =0.0953-2.24\times 10^{-6}T-2.560\times 10^{-7}T^{2},$$

where T is the working mass’s thermodynamic temperature.

The viscosity equation was activated together with the k-ε turbulence model. In the porous enhanced structure, copper served as the solid substrate and water as the working fluid. The copper foam skeleton adopted copper material, and superhydrophobic SiO_2_ nanofibers were configured according to 100 nm superhydrophobic SiO_2_ nanofibers. The porous medium model was implemented with predefined pore parameters. Upon completion of the above settings, boundary conditions were assigned: the model top was specified as a pressure outlet with a steam backflow temperature of 373.15 K and a backflow fraction of 1, while the bottom of the heat-conducting column was set as a heat source. New planes and monitoring points were established in the computational domain to generate contour plots. A reduced time step was applied to improve calculation precision. The iteration ran to convergence, followed by saving of data and files. Subsequent post-processing was conducted to extract parameters and analyze the final simulation outcomes.

The numerical model was solved iteratively. Systematic data collation and comparative analysis were implemented, and the corresponding research findings are presented as follows.

**Figure 15:**
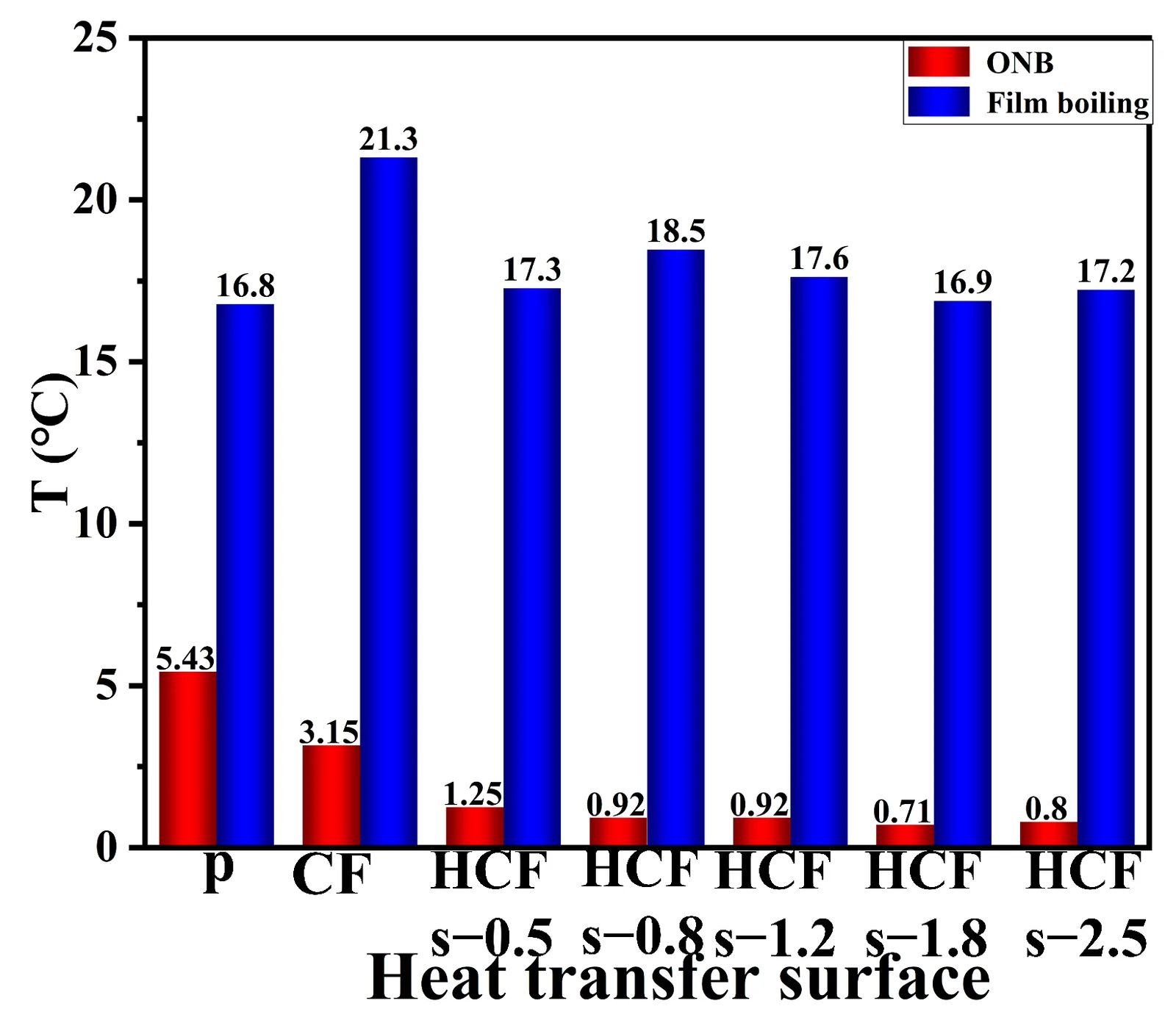
ONB point and film boiling temperature obtained from the simulation.

Figure 15 illustrates the simulated onset and film boiling temperatures. The red areas represent the onset boiling temperature of HCFs heat transfer structures with different deposition amounts. The surface with 1.8 mg of HCFs deposition shows the minimum temperature of 0.71 °C; a reduction of 4.72 °C relative to the copper surface. The blue areas stand for the film boiling temperature. The ΔT for film boiling of all the HCFs structures is obviously decreased compared with the hydrophilic copper foam, showing a high consistency with experimental results.

**Figure 16:**
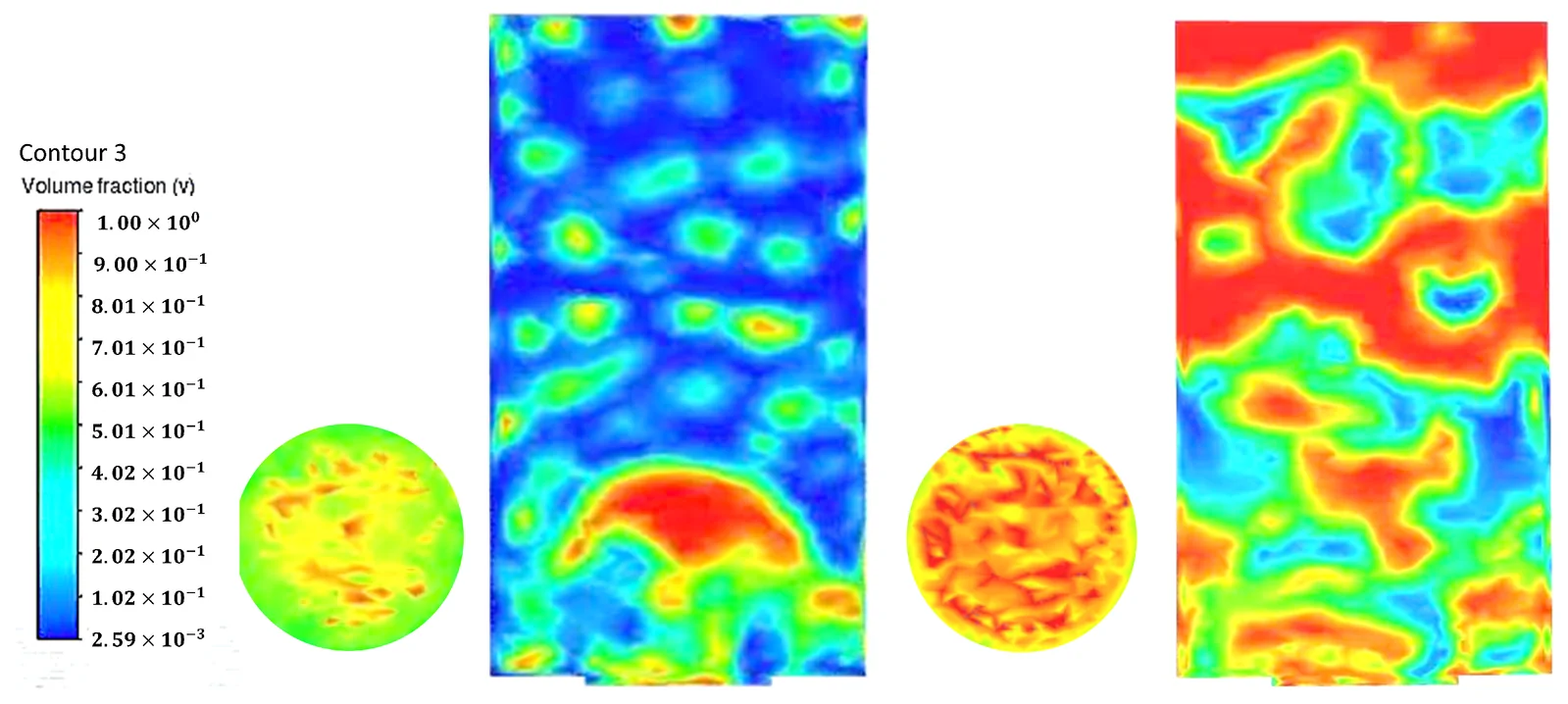
Bubble diagram of HCFs−1.8 heat transfer surfaces: bubble simulation growth.

Figure 16 presents the simulated bubble growth behavior of the micro/nanocomposite structure with 1.8 mg of HCFs deposition. Bubbles nucleate within pores and depart once boiling initiates. With increasing surface temperature, growing bubbles expand and coalesce on the heat transfer surface. A continuous vapor film is subsequently formed to completely occupy the pores, and the system finally enters the film boiling regime.

**Figure 17:**
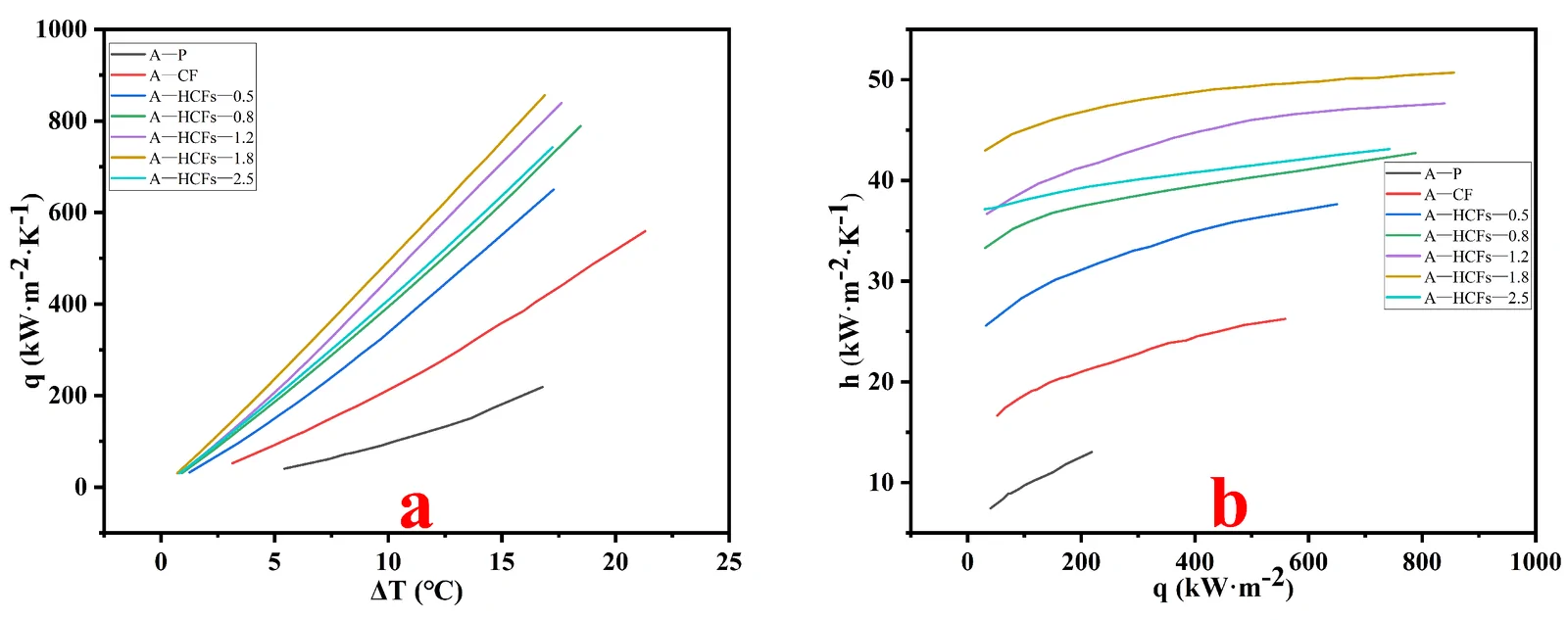
Simulation results of HCFs composite structure. (**a**) The calculated variation curves of q with ∆T; (**b**) the calculated variation curves of h with q.

Figure 17 plots the simulated pool boiling performance of the HCFs micro/nanocomposite structure. Figure 17a illustrates how heat flux varies with wall superheat. For all tested surfaces, heat flux increases monotonously with wall superheat. Under identical wall superheat, heat flux rises along with HCFs deposition amount and peaks at 1.8 mg.

Figure 17b depicts the variation in the heat transfer coefficient against heat flux. The coefficient rises sharply at low heat flux and increases mildly at high heat flux. At the same heat flux, higher HCFs loading corresponds to a larger heat transfer coefficient, with the optimal value observed at 1.8 mg of deposition.

**Figure 18:**
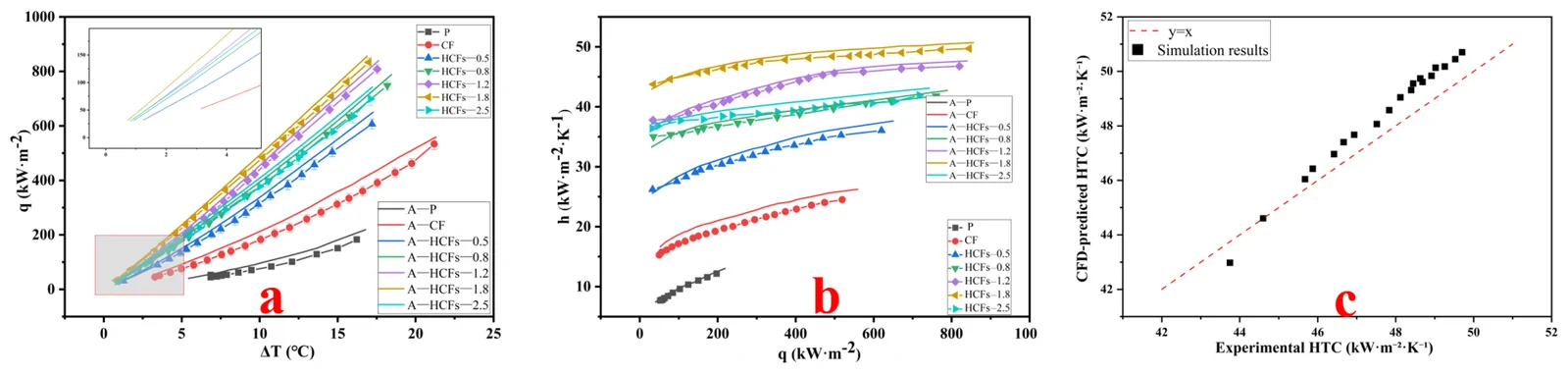
Comparison of experimental results with calculated results on HCFs composite structure. (**a**) The calculated variation curves of q with ∆T; (**b**) the calculated variation curves of h with q; (**c**) parity plot of experimental versus CFD-predicted HTC.

Figure 18 shows the comparison between experimental measurements and numerical predictions for the HCFs micro/nanocomposite structure. Model parameter calibration and mesh refinement improve the numerical simulation reliability, leading to reasonable consistency between calculated and experimental results. Figure 18a,b illustrate the HTC curve comparisons between experimental and simulated results for different samples, which exhibit similar variation tendencies.

To quantitatively assess the prediction performance of the present CFD model, parity plot analysis is provided in Figure 18c. Corresponding statistical metrics are annotated inside the figure, with an average absolute relative error (AARE) of 1.63%. It should be noted that several simplifications are adopted in the treatment of wall nucleation and phase-change interfaces within the numerical framework. The good agreement of the macroscopic HTC is therefore achieved under the experimental conditions investigated in this work, while further improvement in microphysical description is still required for broader-condition applications.

Experimental and numerical results reveal that the HCFs sample with 1.8 mg of superhydrophobic SiO_2_ nanofiber deposition possesses the best overall heat transfer performance, and the 1.2 mg sample shows the fastest performance improvement. The HCFs—1.8 structure dramatically elevates vaporization core density. The superhydrophobic SiO_2_ nanofibers lower the bubble nucleation energy barrier. This sample yields the maximum critical heat flux and heat transfer coefficient in the present work, and provides the most effective enhancement for pool boiling.

## 5. Conclusions

In this study, the pool boiling heat transfer performance of copper foam surfaces with different superhydrophobic SiO_2_ nanofiber deposition amounts was tested, and the heat transfer mechanism of this composite was analyzed. The main conclusions are as follows:

(1) In this study, micro/nanocomposite heat transfer surfaces were constructed on copper foam substrates via hydrothermal deposition of electrospun superhydrophobic SiO_2_ nanofibers. Morphological characterizations confirm satisfactory pore interconnectivity and homogeneous nanofiber coverage across the modified substrates; both HCFs—1.8 and HCFs—2.5 achieve robust superhydrophobicity. The synergistic effect of porous copper foam and deposited SiO_2_ nanofibers lowers bubble nucleation energy barriers and enriches surface evaporation sites, laying a structural foundation for improved boiling heat transfer performance.
(2) Pool boiling experiments reveal that the superhydrophobic SiO_2_ nanofiber coating imparts a substantial improvement in pool boiling performance, with the deposition loading imposing a prominent modulatory effect on thermal–hydraulic characteristics. Of all fabricated specimens, HCFs—1.8 yields the superior overall boiling performance. Against the reference smooth copper baseline, HCFs—1.8 suppresses boiling superheat and achieves concurrent augmentation of critical heat flux (CHF) and the heat transfer coefficient (HTC). The favorable bubble dynamic characteristics facilitate intensified interfacial thermal transport, which constitutes the dominant mechanism responsible for the attained boiling heat transfer enhancement.
(3) VOF-based CFD simulations for pool boiling on the composite structures were implemented in Fluent, and grid independence tests were conducted to select the optimal numerical setup. Satisfactory consistency between simulation outputs and experimental measurements validates the reliability of the established model and parameter settings. The proposed numerical framework is capable of accurately reproducing boiling thermal behaviors of superhydrophobic composite structures, which offers a dependable methodological reference for subsequent numerical investigations and mechanistic exploration of analogous functional boiling surfaces.

## Figures and Tables

**Table 1:** Heat transfer surface parameters of HCFs composite structure.

Named	Substrate Material	Sedimentary Material	Sedimentation/mg
P	Cu	——	——
CF	copper foam	——	——
CFSNFs—2.5	copper foam	SiO_2_ nanofibers	2.5
HCFs—0.5	copper foam	Superhydrophobic SiO_2_ nanofibers	0.5
HCFs—0.8	copper foam	Superhydrophobic SiO_2_ nanofibers	0.8
HCFs—1.2	copper foam	Superhydrophobic SiO_2_ nanofibers	1.2
HCFs—1.8	copper foam	Superhydrophobic SiO_2_ nanofibers	1.8
HCFs—2.5	copper foam	Superhydrophobic SiO_2_ nanofibers	2.5

Notes: P represents the plain smooth copper surface; CF denotes copper foam, where C stands for Cu and F for foam. HCFs refers to superhydrophobic copper-foam-based SiO_2_ nanofibers, where H indicates superhydrophobic modification, CF is copper foam, and S denotes SiO_2_ nanofibers. The numbers following the hyphen represent the sedimentation mass in mg.

**Table 2:** Comparison of boiling performance and preparation methods with recent studies.

Manufacturing Technique	ONB	CHF	HTC	Main Limitations
Physical deposition [41]	73%	56%	52%	Weak bonding force, difficult to obtain porous structure
Selective laser melting [42]	60%	17%	67%	High cost, prone to microcracks
Electrophoretic deposition technology [43]	76%	83%	59%	Dependent on conductive materials, sensitive to parameters
Electrospun fiber deposition	88%	362%	341%	High cost, requires calcination

## Data Availability

The dataset is available upon request from the authors.
